# *CD48*, *CD69*, and *TIGIT* as diagnostic biomarkers for primary Sjögren’s syndrome: an integrated machine learning and multi-disease discrimination validation study

**DOI:** 10.3389/fimmu.2025.1700831

**Published:** 2025-12-16

**Authors:** Linlin Xu, Pingping Jiang, Yang Xiao, Hongling Wang, Hongbo Liu, Huoying Chen

**Affiliations:** 1 Department of Laboratory Medicine, The Second Affiliated Hospital of Guilin Medical University, Guilin, China; 2 Department of Emergency Medicine, The Second Affiliated Hospital of Guilin Medical University, Guilin, China; 3 The First Affiliated Hospital of Guilin Medical University, Guilin, China; 4 Guangxi Health Commission Key Laboratory of Glucose and Lipid Metabolism Disorders, Guangxi Key Laboratory of Metabolic Reprogramming and Intelligent Medical Engineering for Chronic Diseases, Guangxi Key Laboratory of Multimodal Biomarkers and Precision Diagnosis, Guilin Medical University, Guilin, China

**Keywords:** bioinformatics, machine learning, primary Sjögren’s syndrome, biomarkers, diagnostic model

## Abstract

**Background:**

Primary Sjögren’s syndrome (pSS) is a chronic systemic autoimmune disorder. However, current diagnostic methods remain limited, necessitating the exploration of non-invasive diagnostic markers with higher specificity.

**Methods:**

This study integrated two GEO expression datasets to identify differentially expressed genes (DEGs) specific to pSS (distinct from SLE) and applied LASSO, XGBoost, RF, and SVM-RFE algorithms to screen candidate genes. Correlation and interaction network analyses were performed, followed by construction and validation of a diagnostic nomogram. The model’s differential diagnostic ability was validated in IgG4-RD, RA, SLE, and SSc cohorts. Additionally, candidate genes and the diagnostic model were experimentally validated using RT-qPCR in clinical samples.

**Results:**

Three candidate genes (*CD48*, *CD69*, and *TIGIT*) were identified, showing significant upregulation in pSS (individual AUC > 0.80). The combined diagnostic model achieved an AUC of 0.924, with AUC > 0.90 in validation sets, efficiently distinguishing pSS from IgG4-RD, RA, SLE, and SSc. RT-qPCR confirmed their high expression in pSS, with the model yielding AUC 0.875 (accuracy/precision > 0.85). Notably, combining these candidate genes with erythrocyte sedimentation rate (ESR) and C-reactive protein (CRP) yielded an AUC of 0.876 and a specificity of 83.3%, outperforming conventional markers such as ANA, anti-SSA, and anti-SSB antibodies.

**Conclusions:**

*CD48*, *CD69*, and *TIGIT* were identified as potential diagnostic markers for pSS. The combined model significantly enhanced diagnostic accuracy and effectively differentiated pSS from other autoimmune conditions. Integration with ESR/CRP substantially improved specificity compared to conventional serological markers.

## 1 Introduction

Primary Sjögren’s syndrome (pSS) is a systemic autoimmune disorder (1) characterized primarily by sicca symptoms (xerostomia and xerophthalmia) (2, 3). Progressive involvement of extraglandular organs (e.g., renal, pulmonary, gastrointestinal, and neurological systems) frequently occurs (4), resulting in multisystem damage that substantially impairs quality of life (5–8). The diagnosis of pSS remains challenging due to its insidious onset and non-specific early manifestations. Current serological markers, including antinuclear antibody (ANA), anti-SSA, anti-SSB, and anti-Ro-52 antibodies, are further limited by suboptimal sensitivity and specificity (9, 10). Significant clinical overlap with other autoimmune diseases, such as systemic lupus erythematosus (SLE), rheumatoid arthritis (RA), systemic sclerosis (SSc), and IgG4-related disease (IgG4-RD), frequently leads to misdiagnosis or diagnostic delays (11, 12). These diagnostic uncertainties often result in inappropriate therapeutic interventions, which not only delay appropriate pSS management but may also allow core sicca symptoms to progress. In severe cases, irreversible damage to exocrine glands, including salivary and lacrimal glands, may occur, adversely affecting long-term patient prognosis. Although labial gland biopsy remains the diagnostic gold standard, its invasive nature carries risks of infection and bleeding, contributing to limited patient acceptance (13). Therefore, the identification of non-invasive diagnostic biomarkers with high sensitivity and specificity represents a critical unmet need in clinical practice.

To address these diagnostic challenges, the identification of non-invasive biomarkers has been pursued. Recent studies have explored autoantibodies such as anti-calreticulin, anti-PDIA3, and anti-AQP5, which may aid in diagnosing seronegative pSS (14, 15). In addition, multi-omics technologies have identified potential biomarkers, including ferroptosis-related genes (*PARP9*, *PARP12*, *PARP14*) and inflammation markers (*LY6E*, *EIF2AK2*, *IL15*, *CXCL10*) (16, 17). Metabolomic analyses have further revealed dysregulated metabolites in pSS patient samples, offering additional diagnostic insights (18, 19). However, most existing studies focus on single or limited markers, and their reliability is often constrained by insufficient validation across diverse cohorts and disease controls. Moreover, the construction of integrated diagnostic models that combine biomarkers with clinical indicators has been underexplored, limiting their clinical applicability.

Leveraging bioinformatics and machine learning (ML), screening of disease-related biomarkers from complex biological data has become central to life-science research (20, 21). These approaches provide novel research frameworks and technical tools for early disease diagnosis, precision therapy, and prognosis assessment. Thus, integrating bioinformatics with ML not only provides innovative insights for pSS diagnosis but also strengthens its clinical translation.

Based on this research background, an integrated bioinformatics and machine learning approach was employed to systematically develop and validate a diagnostic model for pSS utilizing a specific combination of *CD48*, *CD69*, and *TIGIT* genes. To our knowledge, this represents the first comprehensive validation of this particular gene signature across multiple GEO datasets and independent clinical cohorts encompassing relevant autoimmune disease controls, including systemic lupus erythematosus, rheumatoid arthritis, and systemic sclerosis. The established model demonstrates robust diagnostic performance and discriminative capacity, showing potential as a novel non-invasive tool for early pSS detection and differentiation from other autoimmune conditions, with substantial implications for clinical application.

## 2 Methods

### 2.1 Datasets acquisition and processing

**Figure 1:**
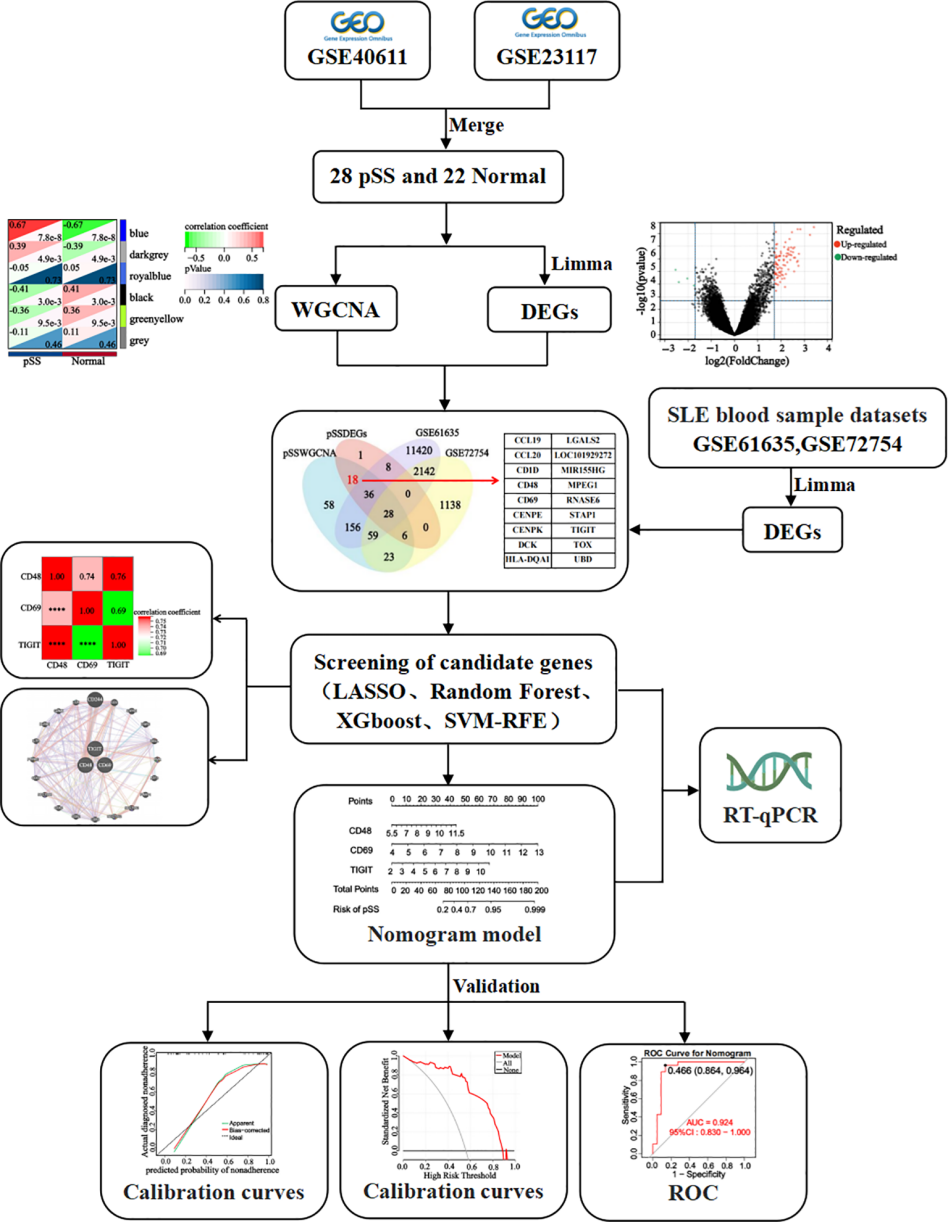
Flow chart of this study design.

The research flowchart is illustrated in **Figure 1**. Four gene chip datasets (GSE40611, GSE23117, GSE127952, and GSE84844) were downloaded from the Gene Expression Omnibus (GEO). Concurrently, their corresponding clinical data and platform annotation files were retrieved (**Supplementary Table 1**). Gene annotation and data normalization were performed using the Sangerbox online tool (http://sangerbox.com) (22). Probe IDs were converted to gene symbols based on platform-specific annotation files, and all probes lacking assigned gene symbols were removed. For genes mapped by multiple probes, a single representative expression value was derived by averaging. To enhance cross-sample comparability, a log_2_ transformation was applied to genes with expression values exceeding 50 in the dataset, generating the gene expression matrix. Subsequently, the insilicoMerging package in R (23) was employed to integrate GSE40611 and GSE23117 datasets. The ComBat method (24) was then applied to correct batch effects within the merged data. To evaluate the efficacy of batch effect correction, data distributions were visually inspected using box plots, density plots, and UMAP plots (**Supplementary Figure 1**). The combined dataset included a total of 28 patients with pSS and 22 healthy controls’ samples. The final normalized and batch-corrected expression matrix was used for subsequent screening of potential diagnostic biomarkers for pSS.

### 2.2 Screening of candidate genes

#### 2.2.1 Screening of differentially expressed genes

The limma package (25) in R 4.3.3 was employed to perform differential expression analysis on the integrated dataset. DEGs were identified using thresholds of |log_2_FC| >1.7 and adjusted *P* < 0.05. To visualize the results, volcano plot was generated. These identified DEGs were subsequently utilized for further screening and analysis.

#### 2.2.2 Identification of hub module genes

Weighted gene co-expression network analysis (WGCNA) was performed using the WGCNA package (26) in R 4.3.3 to identify hub modules and genes associated with pSS clinical characteristics. First, the optimal soft threshold was determined via scale independence and average connectivity assessment (screening criteria: power fit index ≥ 0.80 or average connectivity < 100). Next, a minimum module size of 30 genes was set, and the gene dendrogram was partitioned into modules using dynamic tree cutting to derive biologically meaningful co-expression modules. Highly similar modules were then integrated based on module eigenvector correlation analysis (merge threshold = 0.25). Through module-trait association heatmap analysis, hub modules significantly correlated with pSS clinical groupings (*P* < 0.05) were identified. Genes with Gene Significance (GS) > 0.1 and Module Membership (MM) > 0.3 were screened from these modules as hub genes for subsequent analysis, establishing their relevance to pSS clinical traits.

#### 2.2.3 Identification of the differential gene set

Using the Venn diagram tool provided by Xiantao Academic Platform (https://www.xiantaozi.com), the DEGs of the integrated dataset were intersected with the WGCNA hub module genes to screen pSS-related candidate genes. Given the shared pathogenesis and overlapping differentially expressed genes between pSS and systemic lupus erythematosus (SLE) (27–29), to identify pSS-specific markers, the intersected genes were further analyzed using Venn diagrams with the DEGs from SLE datasets (GSE61635 and GSE72754). This process yielded a gene set that did not overlap with SLE DEGs. These genes will serve as input features for subsequent machine learning algorithms, enabling the identification of candidate diagnostic markers for pSS.

#### 2.2.4 Machine learning algorithms screen candidate genes

The integrated dataset was randomly partitioned into training and testing sets at a 7:3 ratio. Multiple machine learning algorithms, including Least Absolute Shrinkage and Selection Operator (LASSO), Random Forest (RF), eXtreme Gradient Boosting (XGBoost), and Support Vector Machine Recursive Feature Elimination (SVM-RFE), were implemented on the training set. Optimal hyperparameters for each model were selected via cross-validation performed exclusively on the training data. Model generalization performance was subsequently evaluated on the independent testing set to ensure predictive robustness and prevent overfitting. Specifically, the glmnet package (30) was used to perform LASSO regression analysis for variable selection and regularization; the XGBoost algorithm was implemented via the xgboost package (31) to optimize feature importance assessment; the randomForest package (32) was applied to execute the Random Forest algorithm and analyze gene contribution; and the SVM-RFE algorithm was run using the e1071 package. An intersection operation was performed on the screened feature genes to identify overlapping genes as candidate markers for pSS, leveraging the complementary strengths of these algorithms. Future studies will focus on elucidating the biological associations between these candidates and pSS, characterizing their expression patterns, validating their diagnostic performance, and evaluating their clinical utility.

### 2.3 Correlation analysis and interaction network analysis of candidate genes

To investigate the expression correlation of candidate genes, this study employed correlation analysis on the integrated dataset’s expression matrix to characterize their co-expression patterns.

The GeneMANIA database (http://www.genemania.org) (33) was used to construct gene interaction networks for candidate genes, predicting their associations in physical interaction, co-expression, functional prediction, subcellular co-localization, genetic interaction, pathway enrichment, and shared protein structural domains.

### 2.4 Validation of candidate genes and diagnostic model

#### 2.4.1 Verification of the expression levels and diagnostic efficacy of candidate genes in the combined dataset

The Xiantao academic tool (https://www.xiantaozi.com) was used to generate expression box plots of candidate genes, visualizing their differential expression in the integrated dataset. To evaluate the diagnostic utility of these genes, we performed receiver operating characteristic (ROC) curve analysis. By calculating the area under the curve (AUC) and its 95% confidence interval (CI), we quantified the diagnostic performance of individual genes. ROC curves were plotted using the pROC package (34) in R 4.3.3.

#### 2.4.2 Construction and evaluation of nomogram model

A nomogram, a visualization tool for predictive models based on multivariate regression analysis, was used to integrate multiple diagnostic factors for pSS. The rms package in R 4.3.3 was employed to construct a binary logistic regression model, with regularization (L2 penalty) applied to prevent overfitting. Model calibration was evaluated using a calibration curve generated by the same rms package, which assessed the consistency between predicted and observed outcomes. Decision curve analysis (DCA) was performed using the rmda package to calculate net benefit and evaluate clinical utility. Finally, a ROC curve was plotted with the pROC package to assess the nomogram’s diagnostic efficacy, supplemented by evaluation metrics including AUC, accuracy, precision, recall, F1 score, and kappa coefficient.

#### 2.4.3 Validation of candidate gene expression and model diagnostic efficacy in the validation sets

Following expression analysis of the integrated dataset and validation of the model’s diagnostic efficacy, the same analytical pipeline was applied to three independent validation sets: the internal validation set (GSE40611), an external salivary gland tissue validation set (GSE127952), and a whole blood sample dataset (GSE84844). This validation strategy was designed to assess the model’s stability and generalizability across different tissue types and platforms.

#### 2.4.4 Capability verification of model differential diagnosis

Given the overlapping pathogenesis among pSS, IgG4-related disease (IgG4-RD), rheumatoid arthritis (RA), SLE, and systemic sclerosis (SSc) (35–38), the established analytical pipeline was applied to four disease datasets(IgG4-RD: GSE40568; RA: GSE68689; SLE: GSE61635; SSc: GSE181549).This cross-dataset validation aimed to identify pSS-specific diagnostic markers with differential diagnostic capabilities, enabling accurate distinction from closely related autoimmune disorders.

### 2.5 General clinical data of clinical samples

A total of 60 patients with pSS, 40 with RA, 20 with SLE, 12 with SSc, and 61 age- and gender-matched healthy controls were recruited from the First Affiliated Hospital of Guilin Medical University between May and September 2024. Demographic data (name, gender, age, hospital admission number) and clinical symptoms were recorded. Laboratory assessments included hematological parameters [white blood cell count (WBC), hemoglobin (HGB), platelet count (PLT), lymphocyte count (LYM)], liver function markers [total bilirubin (TBIL), direct bilirubin (DBIL), indirect bilirubin (IBIL), globulin (GLB)], immunological indices [immunoglobulins (IgM, IgA, IgG), complements (C3, C4), anti-streptolysin O (ASO), C-reactive protein (CRP), rheumatoid factor (RF), erythrocyte sedimentation rate (ESR)], and autoantibodies [antinuclear antibody (ANA), anti-SSA, anti-SSB, anti-Ro-52]. All assays were performed by the Department of Laboratory Medicine according to standardized protocols.

### 2.6 RT-qPCR

Total RNA was extracted from whole blood using the Trizol method. RNA purity and concentration were measured with a BIOFUTURE MD2000D ultramicro spectrophotometer (Shanghai Biofuture Technology, China). Reverse transcription of RNA to cDNA was performed using the Mona two-step RT kit following the manufacturer’s instructions. Reverse Transcription Quantitative Polymerase Chain Reactio (RT-qPCR) was conducted on an ABI7500 system (Thermo Fisher Scientific, USA) with SYBR Green dye (Mona Biotechnology, China), using β-actin as the internal reference gene. Relative gene expression was calculated by the 2^-ΔΔCt^ method.

### 2.7 Statistical analysis

Statistical analyses were performed using IBM SPSS Statistics 25.0 and GraphPad Prism 9.4.1. Categorical data were presented as percentages, while normally distributed continuous data were expressed as mean ± standard deviation ( $\bar{x}$ ± s). Non-normally distributed continuous data were described by median with interquartile range (IQR). The chi-square test was used for between-group comparisons of categorical data. Statistical significance was defined as *P* < 0.05.

## 3 Results

### 3.1 Three candidate genes—*CD48*, *CD69*, and *TIGIT*—were identified

**Figure 2:**
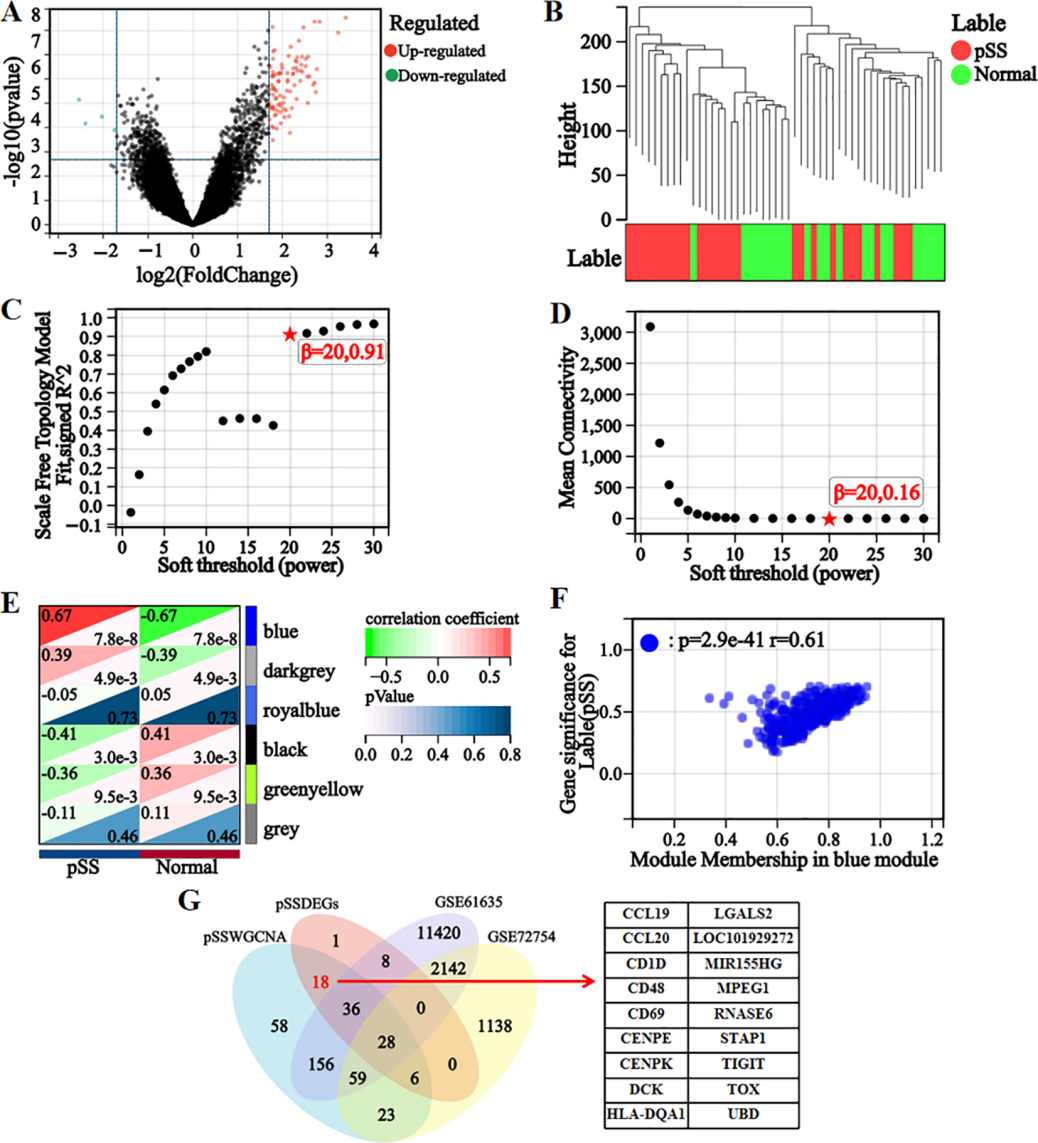
Screening for differential genes. **(A)** Volcano map of differentially expressed genes. **(B)** Dendrogram of 50 samples after clustering. **(C)** Scaled independence plot for soft threshold selection. **(D)** Mean connectivity plot for soft threshold. **(E)** Heatmap of correlations between modules and clinical information. **(F)** Scatterplot of GS-MM correlations in hub modules, where GS represents gene-phenotype correlation and MM represents module characteristic genes. **(G)** The intersection of DEGs in pSS and WGCNA hub module genes was subjected to Venn analysis with DEGs from SLE, yielding differential genes that did not overlap with DEGs in the SLE datasets.

Ninety-seven DEGs were identified in the integrated dataset, comprising 93 upregulated and 4 downregulated genes. These results were visualized through volcano plot analysis (**Figure 2A**), establishing the foundation for subsequent diagnostic marker screening.

Six gene modules were identified by WGCNA (**Figures 2B–E**). The blue module was selected as the hub module based on its strong phenotypic correlation (r=0.61, *P* = 2.9×10^-41^) and statistical significance (*P* < 0.05). 384 hub genes were extracted from this module using screening thresholds of GS > 0.1 and MM > 0.3 (**Figure 2F**).

To enhance the specificity and accuracy of pSS diagnostic markers, the screened pSS DEGs were intersected with the hub genes identified by WGCNA. The resulting gene set was subsequently compared with SLE datasets (GSE61635 and GSE72754), and genes demonstrating overlapping expression patterns were excluded. Through this process, 18 differential genes were identified (**Figure 2G**), which served as inputs for subsequent machine learning analysis to screen characteristic genes.

**Figure 3:**
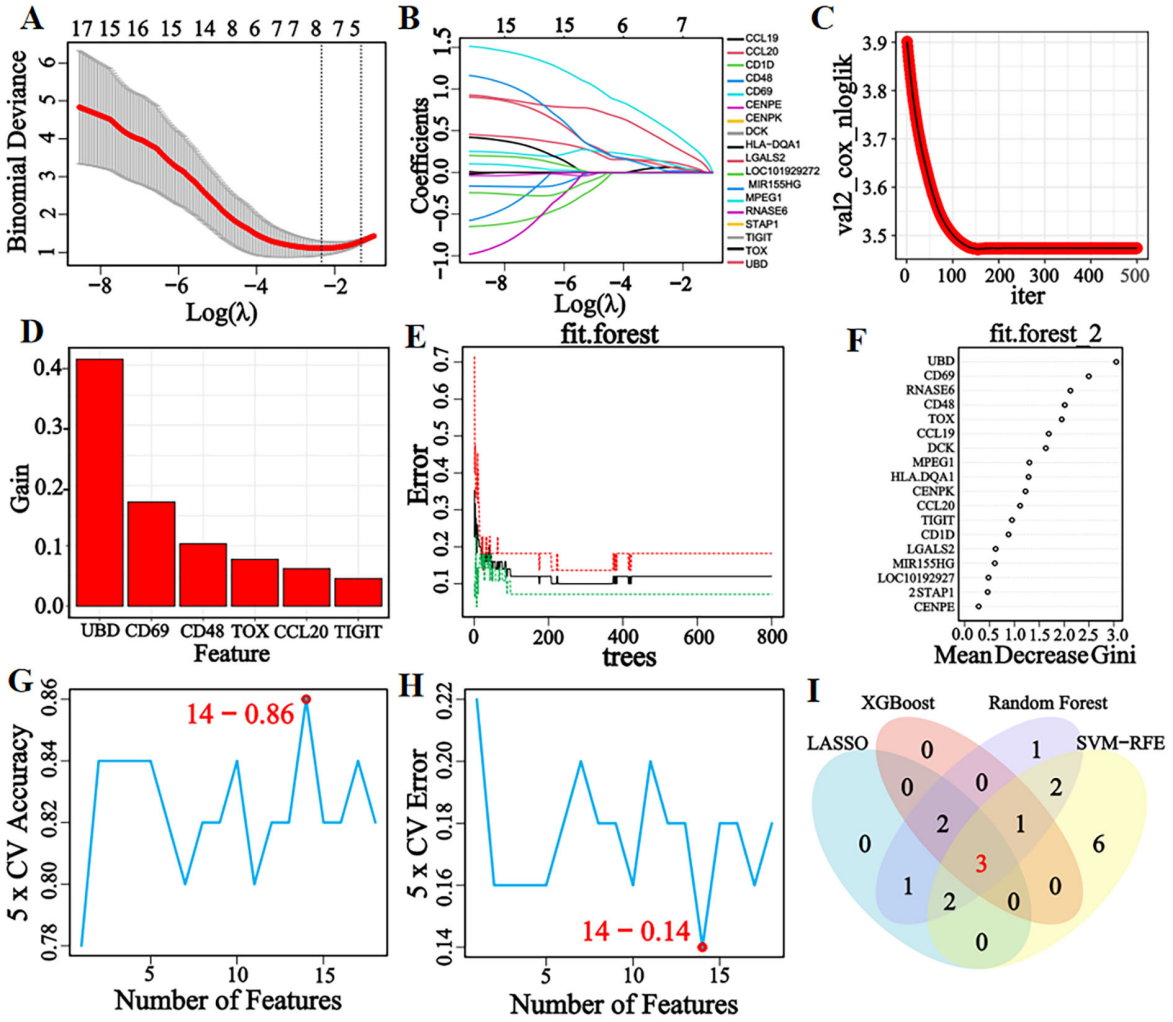
LASSO, XGBoost, RF, and SVM-RFE machine learning algorithms for screening feature genes. **(A)** LASSO regression parameter plot. **(B)** LASSO regression coefficient path plot. **(C)** XGBoost learning curve plot. **(D)** XGBoost feature gene importance histogram, the higher the score, the higher the importance ranking, the greater the contribution to the prediction result. **(E)** The relationship graph between the number of decision trees and error rate in the RF algorithm. As the number of decision trees increases, the error rate of the RF model gradually decreases, and converges to a stable state when the number reaches 425, indicating that the model has achieved a relatively stable performance. **(F)** Gini coefficient results of characteristic genes in the RF algorithm. **(G, H)** Accuracy and error rate curves obtained from 5-fold cross-validation of the SVM-RFE algorithm. When the number of characteristic genes is set to 14, the algorithm achieves an accuracy of 0.86 and an error rate of 0.14. **(I)** Venn diagram of the intersection of feature genes selected by four machine learning algorithms.

Eight, six, twelve, and fourteen feature genes were selected by LASSO, XGBoost, RF, and SVM-RFE algorithms, respectively (**Figures 3A–H**). The intersection of these four gene lists yielded three candidate genes (*CD48*, *CD69*, and *TIGIT*)—as potential diagnostic biomarkers for pSS (**Figure 3I**).

### 3.2 Correlation analysis and interaction network analysis of candidate genes

**Figure 4:**
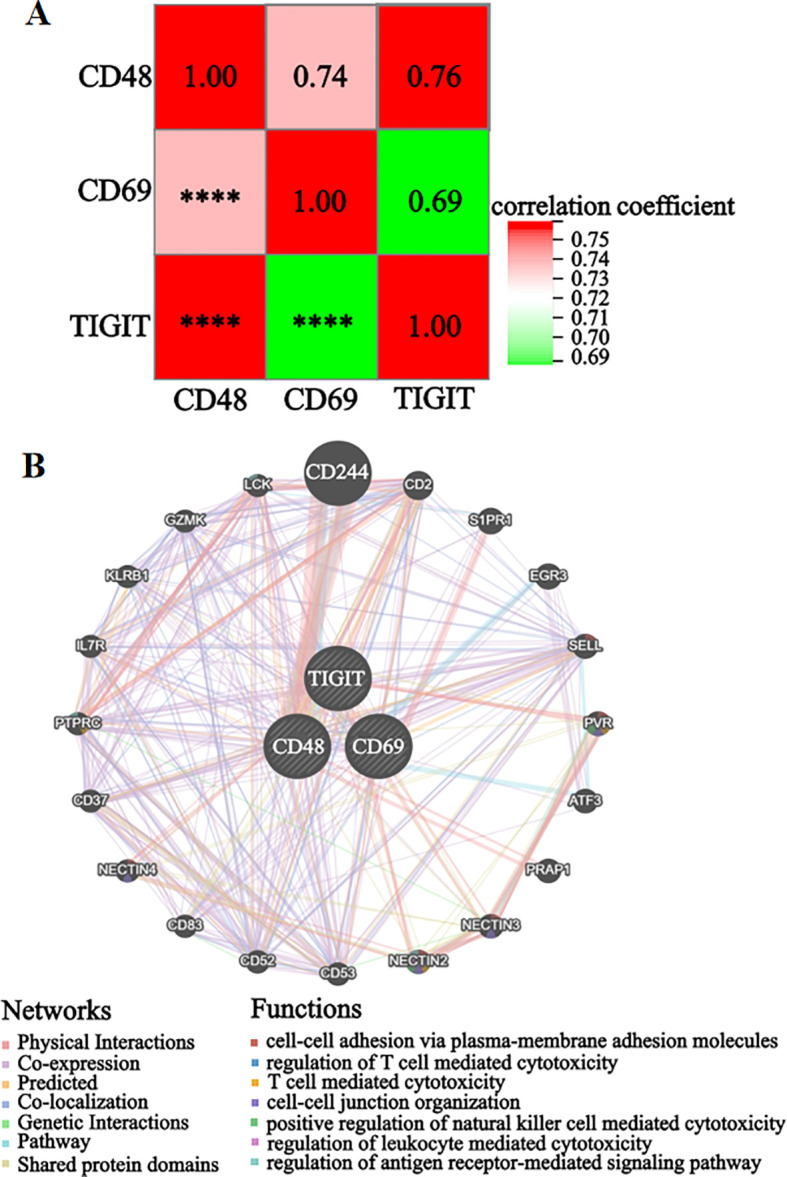
Analysis of the correlation and interaction network of candidate genes. **(A)** Candidate genes correlation heatmap. **(B)** Candidate genes interaction network. ****P < 0.0001.

Correlation analysis of candidate genes based on the integrated dataset’s expression matrix revealed significant positive correlations (cor > 0.69, *P* < 0.001) among the three genes, as shown in the correlation heatmap (**Figure 4A**). The gene interaction network analysis indicated that *CD48*, *CD69*, and *TIGIT* were significantly enriched in immune regulation-related biological processes (*P* < 0.05), including cell adhesion via membrane molecules, T cell-mediated cytotoxicity regulation, cell-cell junction organization, natural killer cell-mediated cytotoxicity activation, leukocyte cytotoxicity regulation, and antigen receptor signaling pathway modulation (**Figure 4B**). These findings suggest that the candidate genes share functional similarities, potentially contributing to pSS pathogenesis by cooperatively regulating immune cell activation and intercellular communication networks.

### 3.3 Successful construction and validation of diagnostic model

**Figure 5:**
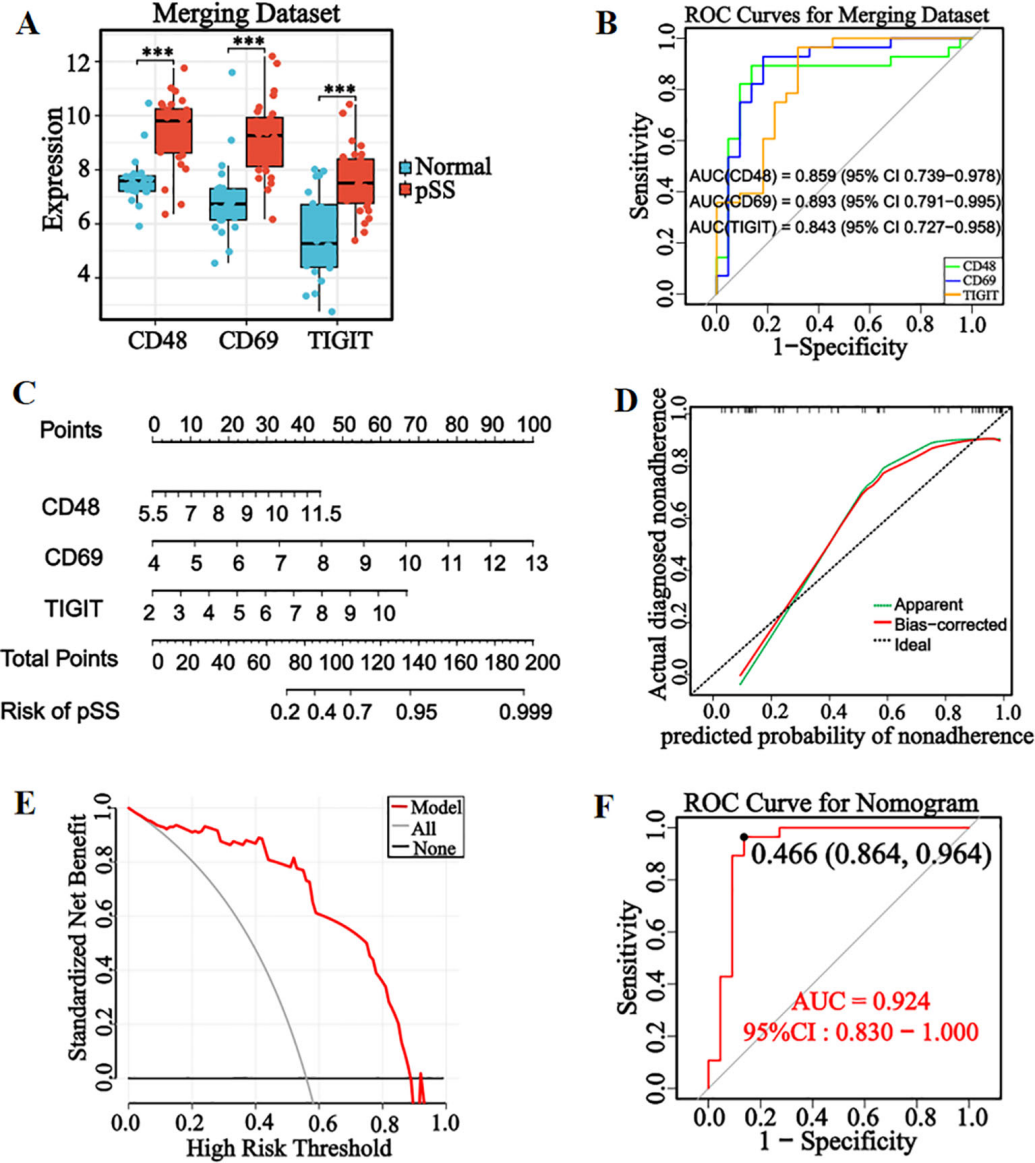
Construction and evaluation of diagnostic model. **(A)** Boxplot of three candidate genes in the merged dataset. **(B)** ROC curves of three candidate genes in the merged dataset. **(C)** Nomogram was constructed from candidate genes.The model assigns weighted scores to each gene and calculates pSS risk probability from the total score. **(D)** Calibration curve. **(E)** Decision curve. **(F)** ROC curve of the nomogram diagnostic model, with optimal cut-off values corresponding to specificity 0.864 and sensitivity 0.964. *P < 0.05, **P < 0.01, ***P < 0.001, ns: No significant difference.

Box plot analysis demonstrated significantly upregulated expression of *CD48*, *CD69*, and *TIGIT* in pSS patients compared with healthy controls (*P* < 0.001, **Figure 5A**). ROC curve analysis showed that all three genes exhibited AUC values > 0.80 (**Figure 5B**), indicating good diagnostic potential for pSS in the integrated dataset.

To enhance the diagnostic and prognostic efficacy for pSS, a nomogram model integrating candidate genes was constructed based on their expression in the integrated dataset (**Figure 5C**). Model evaluation showed that the calibration curve closely matched the ideal curve (**Figure 5D**), demonstrating strong consistency between predicted and observed outcomes. Decision curve analysis (DCA) confirmed significant clinical net benefit across a broad threshold range (**Figure 5E**), while ROC curve analysis revealed an AUC of 0.924 (95% CI: 0.830–1.000), optimal cut-off of 0.466, and corresponding specificity/sensitivity of 86.4%/96.4% (**Figure 5F**). Model performance metrics (**Supplementary Table 2**) showed an accuracy of 88.0%, precision of 83.3%, recall of 0.909, F1 score of 0.870, and Kappa of 0.759, indicating high reliability for clinical decision support in pSS diagnosis.

**Figure 6:**
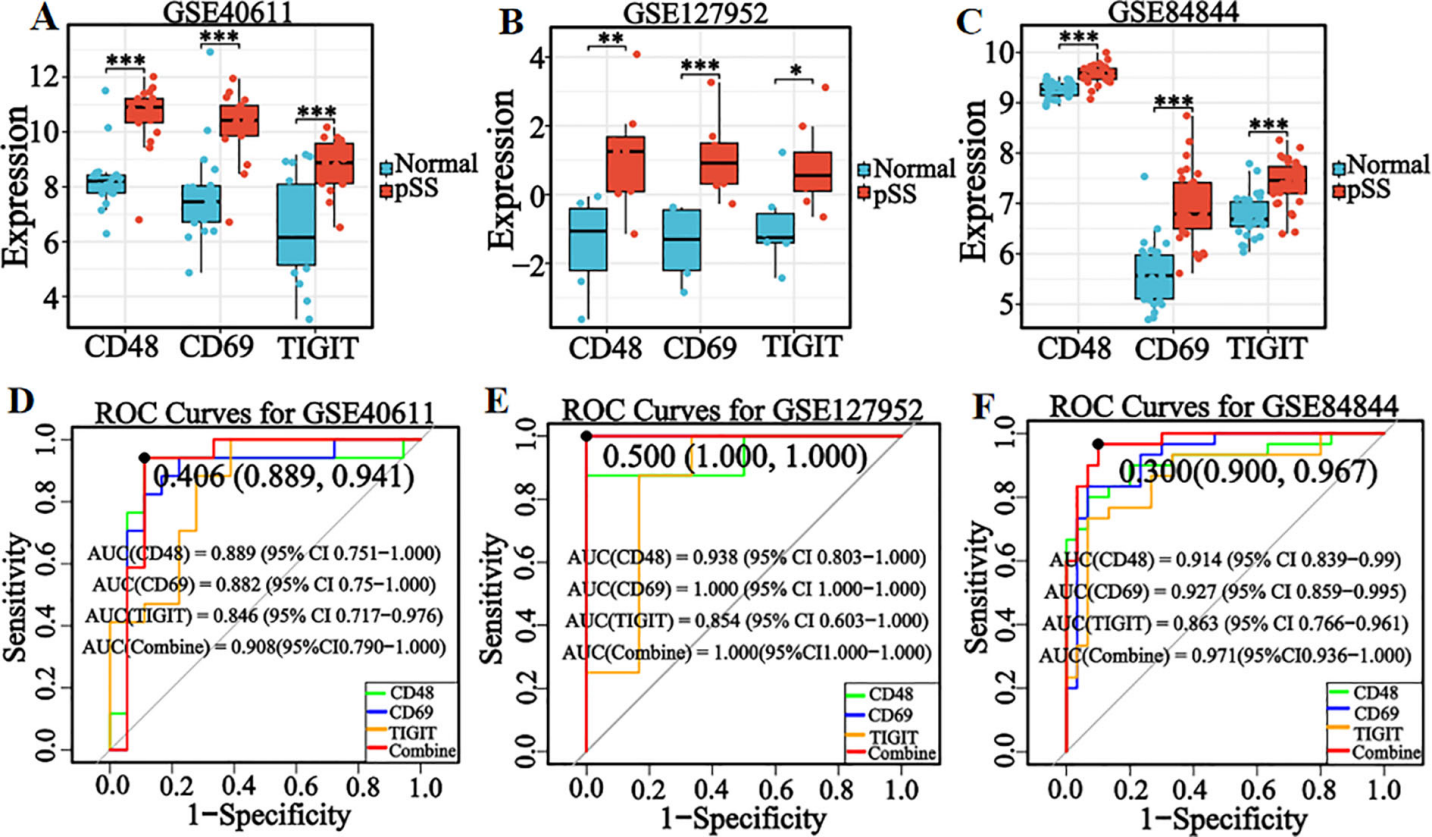
Verification of the expression levels and diagnostic efficacy of candidate genes in internal and external datasets. **(A–C)** Box plots of the expression levels of three candidate genes in the internal validation set (GSE40611), external salivary gland tissue dataset (GSE127952), and external whole blood sample dataset (GSE84844). **(D–F)** ROC curves of three candidate genes in internal validation set (GSE40611), external salivary gland tissue dataset (GSE127952), and external whole blood sample dataset (GSE84844). Optimal cut-offs on the curves are denoted by points, with corresponding specificity and sensitivity values shown in parentheses. *P < 0.05, **P < 0.01, ***P < 0.001, ns: No significant difference.

Box plots (**Figures 6A–C**) showed significantly higher expression of candidate genes in pSS patients than healthy controls across the internal validation set, external salivary gland tissue set, and whole blood sample set (all *P* < 0.05). ROC curve analysis (**Figures 6D–F**) revealed AUC values > 0.80 for individual genes and > 0.90 for the combined diagnostic model. In the external salivary gland tissue validation set GSE127952, the diagnostic model was observed to exhibit perfect discrimination (AUC = 1.00) and recall (1.00), a pattern that could indicate potential model overfitting. The model maintained mean accuracy and precision > 0.85 in all validation sets (**Supplementary Table 3**). These results demonstrate stable diagnostic efficacy of candidate genes across tissue types and platforms, with combined modeling enhancing performance. The consistency of validation outcomes with the integrated dataset analysis further supports their reliability as pSS diagnostic markers.

**Figure 7:**
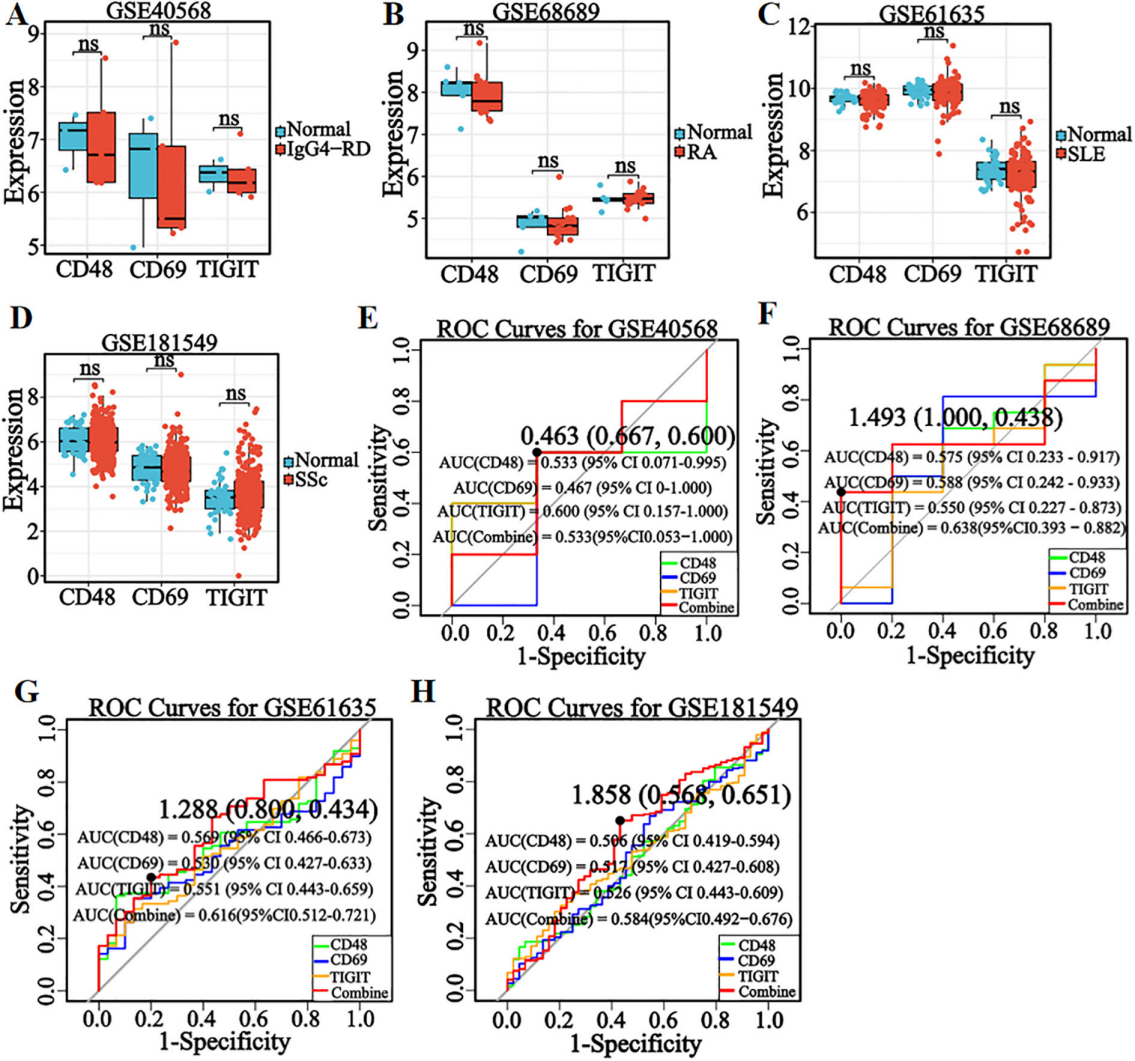
Verification of the expression levels and diagnostic efficacy of candidate genes in the differential diagnosis of diseases. **(A–D)** Box plots of the expression levels of three candidate genes in IgG4-RD (GSE40568), RA (GSE68689), SLE (GSE61635), and SSc (GSE181549) datasets. **(E–H)** ROC curves of three candidate genes in IgG4-RD (GSE40568), RA (GSE68689), SLE (GSE61635), and SSc (GSE181549) datasets. Optimal cut-offs on the curves are denoted by points, with corresponding specificity and sensitivity values shown in parentheses. *P < 0.05, **P < 0.01, ***P < 0.001, ns: No significant difference.

pSS shares similar pathogenesis and clinical features with autoimmune diseases (IgG4-RD, RA, SLE, SSc). To evaluate the differential diagnostic specificity of candidate genes and the nomogram model, relevant disease datasets were downloaded from GEO (**Supplementary Table 4**). Expression levels of *CD48*, *CD69*, and *TIGIT* were visualized using box plots overlaid with scatter plots in IgG4-RD, RA, SLE, and SSc datasets. Results (**Figures 7A–D**) showed no significant expression differences of the three genes between pSS and healthy controls in all four differential diagnosis datasets (all *P* > 0.05), confirming their specificity for pSS.

Given the imbalanced sample distribution in the SSc dataset, the SMOTE algorithm was applied for balancing, followed by diagnostic efficacy evaluation. To validate the model’s differential diagnostic utility, ROC curve analyses were performed in IgG4-RD, RA, SLE, and SSc datasets. Results (**Figures 7E–H**) showed that both single-gene and combined-model AUC values were < 0.70 across all four datasets. Model performance metrics (**Supplementary Table 5**) revealed accuracy and precision < 0.80, with a Kappa coefficient < 0.10. These findings suggest the diagnostic model has some differential diagnostic ability for distinguishing pSS from IgG4-RD, RA, SLE, and SSc.

### 3.4 Validation of expression levels and diagnostic capabilities of candidate genes in clinical samples

**Figure 8:**
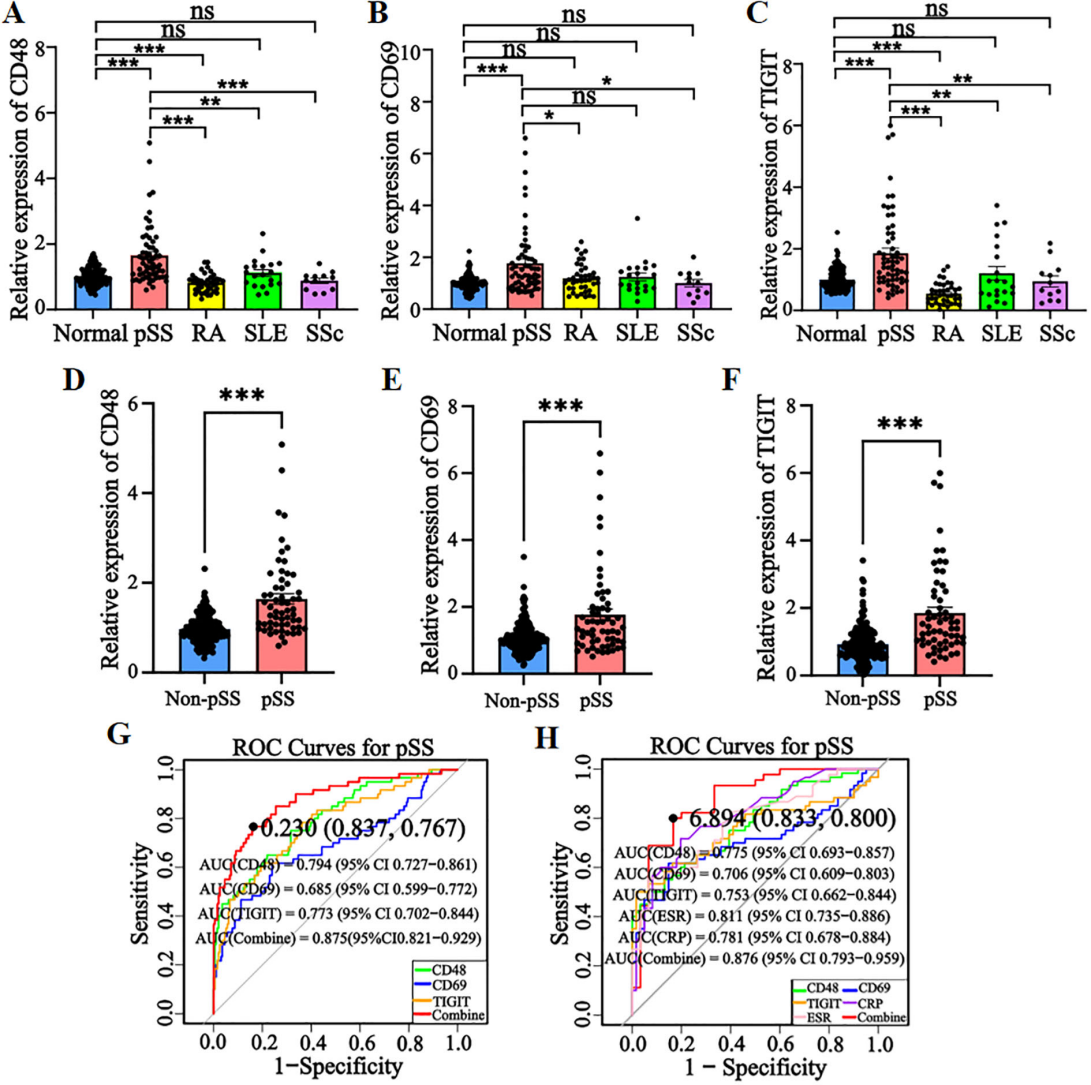
Evaluation of candidate gene expression levels and model diagnostic efficacy in clinical samples. **(A–C)** Bar charts of relative expression levels of *CD48*, *CD69*, and *TIGIT* in healthy controls, pSS, RA, SLE, and SSc groups (RT-qPCR). **(D–F)** Bar charts of candidate gene expression levels in non-pSS controls vs. pSS groups. **(G)** ROC curves of candidate genes in merged non-pSS controls (healthy controls + differential diagnosis diseases) vs. pSS groups. **(H)** ROC curve for the combination of *CD48*, *CD69*, and *TIGIT* with ESR and CRP. The optimal cut-off values on the curve are denoted by points, with corresponding specificity and sensitivity shown in parentheses. *P < 0.05, **P < 0.01, ***P < 0.001, ns: No significant difference.

RT-qPCR analysis showed significantly upregulated expression of *CD48*, *CD69*, and *TIGIT* in peripheral blood from pSS patients compared with healthy controls (*P* < 0.001). In RA patients, *CD48* and *TIGIT* expression was significantly lower than in controls (*P* < 0.001), opposite to the trend in pSS, while *CD69* expression did not differ significantly. No significant differences were observed in SLE or SSc patient samples (**Figures 8A–C**). When healthy controls and differential diagnosis groups (RA, SLE, SSc) were combined into a non-pSS control group, bar chart analysis (**Figures 8D–F**) confirmed marked upregulation of all three genes in pSS patients (*P* < 0.001). These results were consistent with prior bioinformatics analyses, validating the high-expression signature of these candidate genes in pSS peripheral blood.

Age-stratified analyses were performed to address potential confounding by age, given the observed correlation between *CD69*/*TIGIT* expression and age. The results suggested that age did not significantly affect the diagnostic performance of *CD69* and *TIGIT* for pSS (**Supplementary Figure 2**).

ROC curve analysis showed the diagnostic model achieved an AUC of 0.875 (**Figure 8G**), outperforming single-gene diagnostics. The model showed accuracy and precision values both exceeding 0.85 (**Supplementary Table 6**), confirming its high diagnostic efficacy in discriminating pSS from non-pSS. These findings provide a robust experimental foundation for the model’s potential use as a clinical diagnostic marker.

### 3.5 Evaluation of the diagnostic efficacy of candidate gene combined laboratory indicators

Clinical data comparison between pSS patients and healthy controls showed no significant differences in age, gender, WBC, HGB, PLT, LYM, GLB, IgM, IgA, C3, C4,ASO,or RF (all *P* > 0.05). By contrast, pSS patients had significantly higher IgG, ESR, and CRP levels (all *P* < 0.05), and lower total bilirubin, direct bilirubin, and indirect bilirubin levels (all *P* < 0.05), compared with controls (**Supplementary Table 7**).

Based on clinical data of pSS and healthy control groups, we compared the diagnostic efficacy of conventional laboratory indices (with *P* < 0.05 in group comparisons), ANA, anti-SSA, anti-SSB, anti-Ro-52 antibodies, and candidate markers. Metrics including AUC, sensitivity, specificity, and 95% CI were evaluated. Using screening criteria (AUC > 0.70 and sensitivity > 80%), ESR and CRP demonstrated optimal sensitivity among all tested indicators (**Table 1**).

**Table 1:** Comparison of diagnostic efficiency of laboratory detection indicators.

Index	AUC	Sensitivity (%)	Specificity (%)	95% CI
TBIL	0.605	57	68	0.504-0.706
DBIL	0.584	85	35	0.482-0.685
IBIL	0.608	57	67	0.506-0.715
IgG	0.690	87	55	0.561-0.818
ESR	0.816	92	50	0.672-0.960
CRP	0.767	87	58	0.642-0.892
ANA	0.374	87.20	33.30	0.231-0.517
anti-SSA antibody	0.578	74.00	10.00	0.454-0.701
anti-SSB antibody	0.548	22.40	68.00	0.410-0.686
Anti-Ro-52 antibody	0.401	77.60	42.30	0.265-0.535
*CD48*	0.775	75.00	68.40	0.591-0.901
*CD69*	0.706	76.00	61.70	0.599-0.772
*TIGIT*	0.753	68.30	78.30	0.702-0.844
*CD48+CD69+TIGIT*	0.869	85.00	73.00	0.814-0.924
*CD48+CD69+TIGIT*+ESR+CRP	0.876	80.00	83.30	0.793-0.959

Using ESR and CRP as core laboratory indicators, we constructed a combined diagnostic model with *CD48*, *CD69*, and *TIGIT*. ROC curve analysis (**Figure 8H**) showed that the *CD48*+*CD69*+*TIGIT*+ESR+CRP model achieved an AUC of 0.876 with 83.3% specificity, outperforming individual biomarkers and other combinations. In summary, this multi-marker model enhances diagnostic specificity and reliability, demonstrating high clinical utility as a potential preferred diagnostic strategy for pSS.

## 4 Discussion

This study integrated gene expression profiles from a combined dataset with bioinformatics and machine learning approaches to identify three candidate genes (*CD48*, *CD69*, and *TIGIT*) as potential diagnostic markers for pSS. These genes were not only significantly upregulated in pSS patients but also enriched in immune regulation and intercellular communication pathways, suggesting their involvement in pSS pathogenesis via immune cell function modulation. A nomogram model constructed using these genes demonstrated good diagnostic efficacy across internal validation sets, external salivary gland tissues, and whole blood samples, effectively distinguishing pSS from IgG4-RD, RA, SLE, and SSc, which highlighted its broad applicability. RT-qPCR experiments validated the high expression of candidate genes in pSS and confirmed the model’s diagnostic and differential capabilities, supporting its stable accuracy and clinical utility. Notably, RT-qPCR results demonstrated that *CD48*, *CD69*, and *TIGIT* were significantly upregulated in patients with pSS, whereas no significant differences were observed in patients with SLE or SSc compared to healthy controls. This experimental finding is fully consistent with the bioinformatics-based filtering strategy, wherein differentially expressed genes in SLE were deliberately excluded during the candidate selection phase. The RT-qPCR results support the disease-specific upregulation of these three markers in pSS, rather than their nonspecific elevation across other systemic autoimmune conditions. The concordance between the bioinformatic prediction and the validation experiments thereby confirms the accuracy and reliability of the screening strategy employed in this study. It is particularly noteworthy that expression levels of *CD48* and *TIGIT* were significantly lower in patients with rheumatoid arthritis compared to those with pSS. This opposing expression trend is likely reflective of fundamental differences in the underlying immune pathogenesis of these diseases. In pSS, these molecules may be involved in the activation of lymphocytes within exocrine glands; whereas in rheumatoid arthritis, their reduced expression may indicate that inflammatory pathways centered on joint pathology predominate. This reciprocal pattern suggests that these biomarkers hold important diagnostic value for differentiating pSS from rheumatoid arthritis. Moreover, when combined with conventional laboratory indicators, ESR and CRP, the triad of candidate genes demonstrated higher diagnostic specificity than traditional serological markers, including ANA, anti-SSA, anti-SSB, and anti-Ro-52 antibodies. This multi-indicator diagnostic strategy provides a framework for individualized management in pSS patients and may serve as a valuable supplementary approach to existing classification criteria (AECG/ACRationrca.

In recent years, high-throughput sequencing and bioinformatics advancements have made gene expression profiling a pivotal approach for screening pSS biomarkers. For example, a study using the GSE40611 dataset identified 232 DEGs, followed by PPI network analysis to screen over 10 key genes (e.g., *PTPRC*, *CD19*, *CD69*), with RT-qPCR validation consistent with microarray data (39). However, reliance on a single dataset may compromise result generalizability. Another study combined PPI, WGCNA, and LASSO regression to identify *SAMD9L* and *XAF1* as diagnostic markers (40), but single-algorithm dependency (LASSO) introduced bias. A third study used WGCNA to screen core module genes (e.g., *EIF2AK2*, *GBP1*, *PARP12*), but single-method limitations hindered key gene identification (41). In contrast, this study integrated two same-platform datasets via standardized merging, leveraging diverse data sources and combining multiple bioinformatics and machine learning approaches to ensure candidate gene accuracy and reliability.Furthermore, a study identified *IRF9* and *XAF1* as pSS diagnostic markers via GSE51092 dataset analysis (42), but lacked experimental validation, potentially compromising result reliability and clinical utility. Another study combined SVM, LASSO, random forest, and WGCNA to identify 10 key genes from 1,643 DEGs in GEO datasets, confirming expression via immunohistochemistry (43), but did not assess differential diagnostic ability. Additionally, a three-algorithm (ANN, RF, SVM) modeling study found RF had optimal predictive performance (44), but without differential diagnostic or clinical sample validation. In contrast, this study constructed a diagnostic model using candidate genes, validated its diagnostic and differential capabilities in gene expression and clinical datasets, and further verified candidate gene expression, model efficacy, and disease-specific discrimination in clinical samples, ensuring result reliability and clinical applicability.

*CD48* (*BLAST-1/SLAMF2*) is a GPI-linked immunoglobulin superfamily member, widely expressed on T cells, B cells, NK cells, dendritic cells, and monocytes, mediating cell adhesion and activation pathways (45, 46). As an immune co-stimulatory and adhesion molecule, *CD48* contributes to autoimmune disease regulation (47), suggesting a potential role in pSS via immune response modulation. Studies show *CD48* is upregulated in pSS salivary gland tissues, promoting local inflammation through JAK-STAT signaling and immune cell infiltration (48), and is highly expressed in salivary gland B lymphocytes, driving B cell activation (49)—findings consistent with this study. Additionally, *CD48* serves as a serum biomarker of disease activity in pSS (50). Notably, however, its systematic validation as a diagnostic marker for pSS remains unaddressed.

*CD69*, a type II transmembrane glycoprotein, is primarily expressed on T cells, B cells, neutrophils, NK cells, macrophages, and eosinophils. It not only drives T cell activation and proliferation but also facilitates rapid immune cell recognition and regulation. As a pivotal immune system component, *CD69* modulates immune cell function via its unique signal transduction pathways (51). These functions suggest a potential role for *CD69* in the pathogenesis of pSS through immunoregulation. Existing studies have shown that *CD69* participates in B cell activation in pSS and correlates with disease activity (52), aligning with the findings of this study. However, its comprehensive validation as a diagnostic biomarker for pSS remains underexplored.

*TIGIT* (T-cell immunoglobulin and ITIM domain protein) is a co-inhibitory immune checkpoint receptor predominantly expressed on T cells and NK cells. It suppresses dendritic cell and macrophage function by promoting IL-10 secretion and mediating the CD155 signaling pathway, thereby inducing immune tolerance. Current evidence shows *TIGIT* contributes to T cell hyperactivation in pSS, as demonstrated by enhanced CD4+TIGIT+ T cell activity (53), elevated *TIGIT* expression on γδ T cells (54), and reduced *TIGIT* levels on CD14+ monocytes (55). These findings establish a research foundation for *TIGIT* as a potential diagnostic biomarker in pSS.

Therefore, in the pathogenesis of pSS, *CD48*, *CD69*, and *TIGIT* likely interact through multiple mechanisms, including co-activation, immune suppression, and inflammation regulation, to modulate immune cell activation and inflammatory responses. Overexpression of *CD48* and *CD69* may cooperatively enhance immune cell activation and amplify immune responses, whereas elevated *TIGIT* expression may, in turn, partially suppress immune cell hyperactivation and attenuate the production of inflammatory cytokines. This interplay among the three molecules may influence inflammatory responses in pSS by modulating the balance of pro- and anti-inflammatory cytokines. Further studies are warranted to elucidate their precise mechanistic roles in pSS.

Despite the achievements of this study, several limitations remain: (i) The relatively small sample size in GEO datasets may compromise result generalizability. (ii) The absence of autoantibody data from healthy controls precluded direct comparison with the pSS group, hindering comprehensive evaluation of diagnostic markers alongside routine laboratory indices. (iii) The small sample size of the autoantibody-negative pSS subgroup (n=7) limited our ability to perform a robust statistical comparison of *CD48*, *CD69*, and *TIGIT* expression between seronegative and seropositive patients or to investigate their association with autoantibody status. (iv) Although regularized, the diagnostic model exhibited overfitting during external validation in salivary gland tissue datasets, likely due to limited sample size. (v) Experimental validation relied primarily on blood samples, neglecting gene expression profiles in pSS salivary glands. (vi) IgG4-RD, a rare disorder, was not included in differential diagnosis validation due to sample acquisition challenges and resource constraints. (vii) The sample sizes of the SLE and SSc cohorts in the RT-qPCR-based differential diagnosis validation were relatively small. This may limit the statistical power to conclusively establish the discriminative efficacy of the proposed biomarkers against these specific autoimmune diseases. (viii) The observation of a perfect AUC (1.00) for the diagnostic model in the external salivary gland tissue dataset is considered to potentially stem from the limited sample size of this particular validation set, as well as the high tissue specificity of the biomarker expression in the target organ. It should be emphasized that L2 regularization had been implemented during the model training phase to ensure robustness. Future directions should include evaluating the diagnostic efficacy of three biomarkers in large multicenter cohorts; integrating proteomic/epigenomic data to validate marker utility; dissecting the role of candidate genes in pSS pathogenesis and immune cell regulation; and developing pSS diagnostic kits based on candidate diagnostic markers and promoting their clinical application.

## 5 Conclusion

This study successfully screened and validated three pSS diagnostic markers (*CD48*, *CD69*, and *TIGIT*) using bioinformatics and machine learning. These markers showed significant differential expression in pSS patients, close association with disease pathogenesis, and robust independent diagnostic efficacy. The marker-based combined diagnostic model not only improved diagnostic accuracy but also exhibited strong differential diagnostic ability, effectively distinguishing pSS from other autoimmune diseases. Clinical validation confirmed elevated expression of all three biomarkers in pSS patients and replicated the model’s diagnostic and differential capabilities, consistent with bioinformatics predictions. Moreover, combining biomarkers with routine indices (ESR, CRP) further enhanced diagnostic specificity. These findings identify potential pSS biomarkers and provide a solid foundation for developing clinical diagnostic tools.

## Data Availability

The datasets presented in this study can be found in online repositories. The names of the repository/repositories and accession number(s) can be found in the article/**Supplementary Material**.
