# Supplementary material for: CD48, CD69, and TIGIT as diagnostic biomarkers for primary Sjögren’s syndrome: an integrated machine learning and multi-disease discrimination validation study
Source: Front Immunol. 2025 Dec 16;16:1700831. doi: 10.3389/fimmu.2025.1700831 (PMC12747951; doi:10.3389/fimmu.2025.1700831)
Supplement: Supplementary file 1 [file Table1.docx]

Supplementary Material

## 1. Supplementary Figures


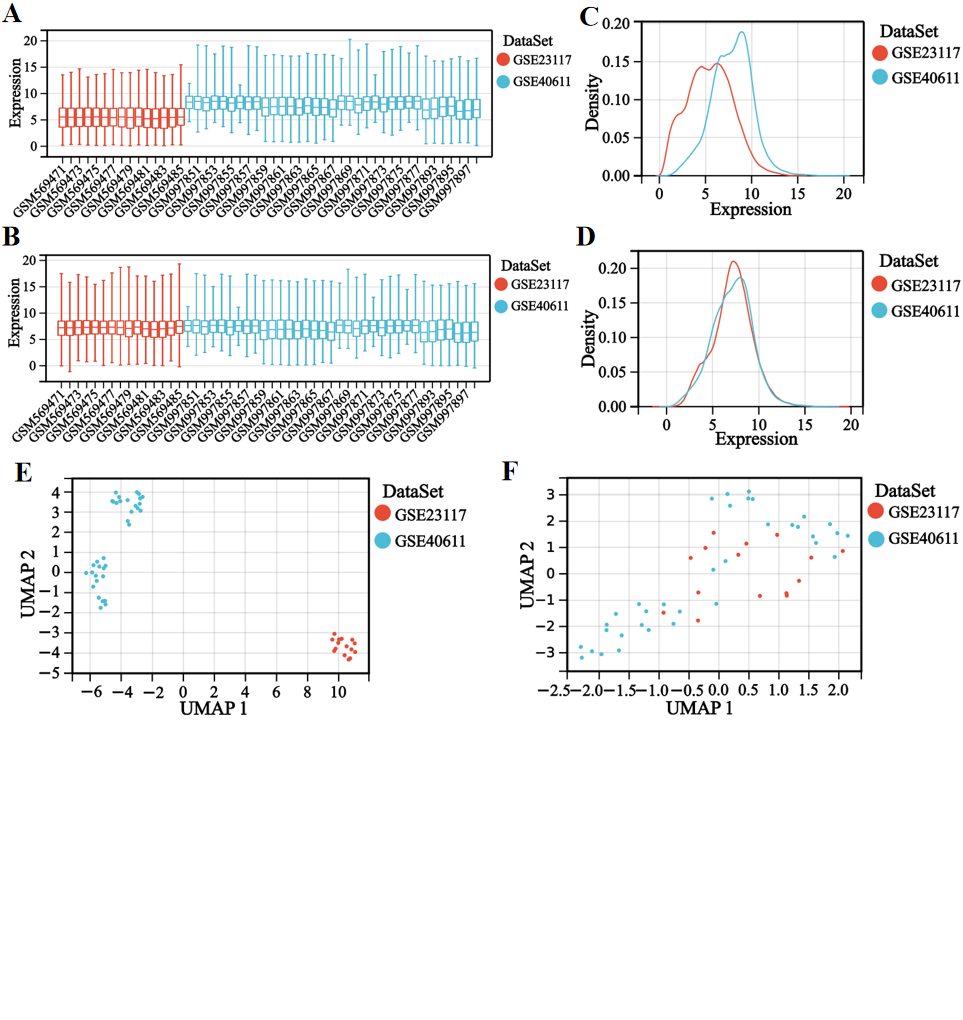


**Supplementary Figure 1 A, B** Boxplots show distinct sample distributions across datasets before and after batch effect removal; after removal, distributions are consistent with medians aligned on a single line. **C, D** Density plots reveal divergent sample distributions among datasets pre-correction and post-correction; post-correction, distributions converge with matched means and variances. **E, F** UMAP plots demonstrate that samples from each dataset form separate clusters before batch effect removal, whereas post-correction, samples intermingle across clusters, confirming effective batch effect elimination.


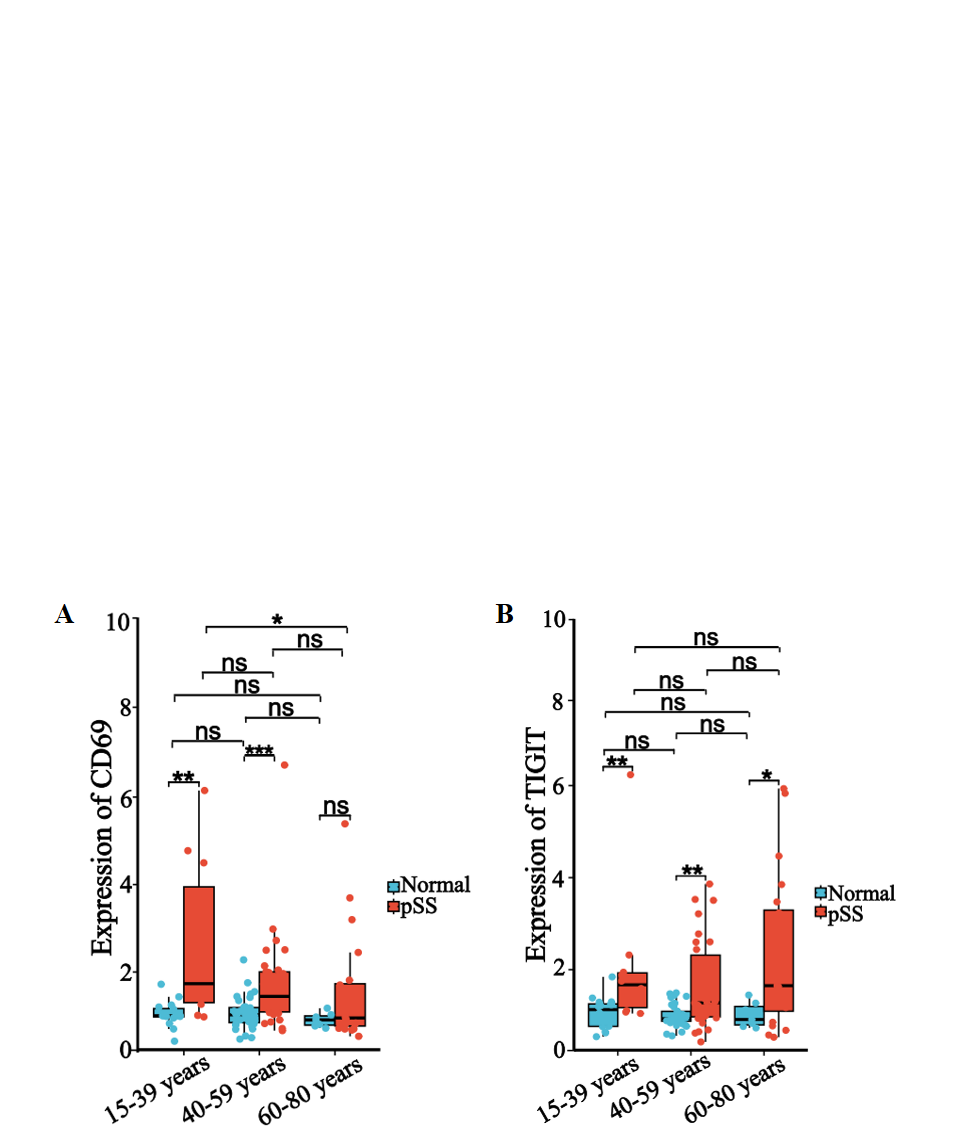


## **Supplementary Figure 2 A** Box plot from age-stratified analysis of *CD69* expression shows that in the pSS group, expression levels are significantly lower in the 60–80 age group than in the 15–39 age group (*P*<0.05), with no statistically significant difference between age groups in the healthy control group (*P*>0.05). **B** Box plot from age-stratified analysis of *TIGIT* expression shows that *TIGIT* expression is significantly higher in pSS patients than in healthy controls across all three age strata (*P*<0.05), with no significant difference between the three strata within pSS patients (*P*>0.05).

## 2. Supplementary Tables

**Supplementary Table 1** The information of the GEO datasets

| **Data set** | **Platform** | **Sample information** | **Application** |
| --- | --- | --- | --- |
| GSE40611 | GPL570 | 35 samples of salivary gland tissues  （17 pSS+18 Normal） | Training set  Internal validation set |
| GSE23117 | GPL570 | 15 samples of salivary gland tissues  （11 pSS+4 Normal） | Training set  Internal validation set |
| GSE127952 | GPL20995 | 14 samples of salivary gland tissues  （8 pSS+6 Normal） | External validation set |
| GSE84844 | GPL570 | 60 samples of whole blood  （30 pSS+30 Normal） | External validation set |

**Supplementary Table 2** The results of evaluation index of nomogram model

| Model | AUC | Accuracy | Precision | Recall | F1 score | Kappa |
| --- | --- | --- | --- | --- | --- | --- |
| Nomogram | 0.924 | 0.880 | 0.833 | 0.909 | 0.870 | 0.759 |

**Supplementary Table 3** Diagnostic performance evaluation of diagnostic model in the validation datasets

| **Data set** | **AUC** | **Accuracy** | **Precision** | **Recall** | **F1 score** | **Kappa** |
| --- | --- | --- | --- | --- | --- | --- |
| GSE40611 | 0.908 | 0.914 | 0.941 | 0.889 | 0.914 | 0.829 |
| GSE127952 | 1.000 | 0.929 | 0.857 | 1.000 | 0.923 | 0.857 |
| GSE84844 | 0.971 | 0.900 | 0.870 | 0.933 | 0.903 | 0.800 |

**Supplementary Table 4** Differential diagnostic disease data set information

| **Data set** | **Platform** | **Sample information** | **Application** |
| --- | --- | --- | --- |
| GSE40568 | GPL570 | 8 samples of salivary gland tissues  （5IgG4-RD+3Normal） | Differential diagnosis dataset |
| GSE68689 | GPL20171 | 21 samples of whole blood  （16RA+5Normal） | Differential diagnosis dataset |
| GSE61635 | GPL570 | 181 samples of whole blood  （141SLE+40Normal） | Differential diagnosis dataset |
| GSE181549 | GPL134957 | 339 samples of forearm skin biopsy tissues  （295SSc+44Normal） | Differential diagnosis dataset |

**Supplementary Table 5** Diagnostic performance evaluation of diagnostic models in differential diagnosis disease datasets

| **Differential diagnosis of diseases** | **AUC** | **Accuracy** | **Precision** | **Recall** | **F1 score** | **Kappa** |
| --- | --- | --- | --- | --- | --- | --- |
| GSE40568 (IgG4-RD dataset) | 0.533 | 0.500 | 0.667 | 0.400 | 0.500 | 0.000 |
| GSE68689 (RA dataset) | 0.638 | 0.714 | 0.778 | 0.875 | 0.824 | 0.087 |
| GSE61635(SLE dataset) | 0.616 | 0.736 | 0.760 | 0.960 | 0.848 | -0.058 |
| GSE181549(SSc dataset) | 0.584 | 0.644 | 0.694 | 0.868 | 0.771 | 0.014 |

**Supplementary Table 6**  Evaluation of diagnostic performance of diagnostic model in clinical samples

| **Model** | **AUC** | **Accuracy** | **Precision** | **Recall** | **F1 score** | **Kappa** |
| --- | --- | --- | --- | --- | --- | --- |
| Model in clinical samples | 0.875 | 0.867 | 0.868 | 0.974 | 0.918 | 0.570 |

**Supplementary Table 7** General laboratory data statistics of pSS and healthy control group

| **Factor** | **Normal(n=61)** | **pSS(n=60)** | ***P*** |
| --- | --- | --- | --- |
| Age ($\overline{x}$±s, years) | 52.03±12.11 | 54.15±12.94 | 0.355 |
| Female, n (%) | 56(92.00%) | 54(90.00%) | 0.776 |
| WBC (*10^9/L) | 5.64(1.61) | 5.885(1.66) | 0.242 |
| HGB (g/L) | 131(14.50) | 130(18.00) | 0.321 |
| PLT (*10^9/L) | 222.0(88.00) | 223.5(65.25) | 0.371 |
| LYM (*10^9/L) | 1.78(0.72) | 1.7605(0.78) | 0.975 |
| TBIL (μmol/L) | 13.06(5.49) | 11.165(4.85) | 0.024 |
| DBIL (μmol/L) | 3.59(2.24) | 3.185(2.27) | 0.043 |
| IBIL (μmol/L) | 9.80(4.40) | 7.99(3.44) | 0.015 |
| GLB (g/L) | 31.00(4.35) | 30.25(7.60) | 0.811 |
| IgM (g/L) | 1.31(0.83) | 1.135(0.68) | 0.857 |
| IgA (g/L) | 3.30(2.46) | 2.94(1.24) | 0.507 |
| IgG (g/L) | 13.53±2.17 | 16.40±5.20 | 0.037 |
| C3 (mg/L) | 1.18(0.18) | 1.445(0.33) | 0.397 |
| C4 (mg/L) | 0.29(0.30) | 0.31(0.12) | 0.693 |
| ASO (IU/L) | 34.00±28.35 | 33.58±29.66 | 0.969 |
| RF (IU/L) | 8.00(10.10) | 8.50(14.90) | 0.678 |
| ESR (mm/h) | 23.00(17.00) | 45.50(13.00) | <0.001 |
| CRP (mg/L) | 1.55(2.61) | 4.98(6.15) | 0.019 |

Counting data were presented as percentages. Normally distributed measurement data were expressed as mean ± SD, while non-normally distributed data were presented as median (interquartile range, IQR).
